# Genomic Sequencing in Neonatal Encephalopathy and Suspected Hypoxic–Ischaemic Encephalopathy: A Systematic Review

**DOI:** 10.3390/genes17080862

**Published:** 2026-07-24

**Authors:** Dario Colacurci, Laura Sarno, Emmanuel Fiore, Francesco Raimondi, Nina Martinelli, Angelo Sirico, Costantino Di Carlo, Giuseppe Bifulco, Maurizio Guida, Giuseppe Maria Maruotti

**Affiliations:** 1 Department of Public Health, University of Naples Federico II, via Pansini 5, 80131 Naples, Italy; nina.martinelli@unina.it (N.M.); costantino.dicarlo@unina.it (C.D.C.); giuseppe.bifulco@unina.it (G.B.); giuseppemaria.maruotti@unina.it (G.M.M.); 2 Department of Neurosciences, Reproductive Science and Dentistry, University of Naples Federico II, via Pansini 5, 80131 Naples, Italy; laura.sarno@unina.it (L.S.); maurizio.guida@unina.it (M.G.); 3 Mother and Child Department, University Hospital Federico II, via Pansini 5, 80131 Naples, Italy; emmanuel.fiore@unina.it; 4 Division of Neonatology, Department of Translational Medical Sciences, Federico II University, via Pansini 5, 80131 Naples, Italy; francesco.raimondi@unina.it; 5 Department of Woman, Child and General and Specialized Surgery, University of Campania “Luigi Vanvitelli”, via Armanni 14, 80138 Naples, Italy; angelo.sirico@unicampania.it

**Keywords:** neonatal encephalopathy, hypoxic–ischaemic encephalopathy, whole-exome sequencing, genetic diagnosis, precision medicine, neonatal seizures

## Abstract

Background: Neonatal encephalopathy (NE) is a major cause of neonatal mortality and long-term neurological disability. Although hypoxic–ischaemic encephalopathy (HIE) is the most common cause, several genetic disorders may mimic or coexist with hypoxic–ischaemic injury. Next-generation sequencing has emerged as a promising diagnostic tool in this setting. This systematic review evaluated the current evidence on genomic sequencing in NE. Material and methods: A systematic review was conducted according to PRISMA 2020 guidelines and prospectively registered in PROSPERO. PubMed/MEDLINE, Embase, and Scopus were searched from inception to June 2026. Eligible studies included neonates (≤28 days) with NE, suspected or confirmed HIE, HIE mimics, or unexplained NE who underwent genomic sequencing. Whole-exome sequencing (WES), whole-genome sequencing (WGS), clinical exome sequencing (CES), rapid genomic sequencing, and targeted next-generation sequencing panels were considered. Study quality was assessed using the Newcastle–Ottawa Scale. Results: Seven studies met the inclusion criteria. Considerable heterogeneity was observed regarding patient selection, sequencing strategies, and reported outcomes. Among diagnostic sequencing studies, diagnostic yield ranged from 23.5% to 53.1%. Pathogenic and likely pathogenic variants were identified in genes associated with developmental and epileptic encephalopathies, metabolic disorders, mitochondrial diseases, and neurodevelopmental syndromes, including SCN2A, KCNQ2, CACNA1A, STXBP1, PTPN11, BCOR, MMUT, COQ2, and GBE1. Genomic sequencing frequently refined or changed the initial diagnosis, improved prognostic assessment and genetic counselling, and, in selected cases, guided disease-specific treatment. One study investigated genetic susceptibility to hypoxic–ischaemic injury rather than diagnostic sequencing. Conclusions: Genomic sequencing provides clinically meaningful diagnoses in a substantial proportion of neonates with unexplained NE or atypical HIE presentations. Current evidence supports integrating genomic sequencing into the diagnostic evaluation of selected infants, although larger prospective studies are needed to define its optimal timing, clinical utility, and cost-effectiveness.

## 1. Introduction

Neonatal encephalopathy (NE) is a clinically defined syndrome of disturbed neurological function occurring during the first days of life in term and near-term infants [1,2]. NE is characterized by distorted consciousness, irregular muscle tone and reflexes, respiratory dysfunction, feeding difficulties, and seizures [1,2,3]. NE is still a major cause of neonatal mortality and long-term neurological disability worldwide, including cerebral palsy, epilepsy, intellectual disability, and developmental delay [4,5,6,7]. For that reason, identifying the underlying cause remains challenging despite advances in neonatal intensive care. Hypoxic–ischaemic encephalopathy (HIE) is the most common cause of NE and represents the main indication for therapeutic hypothermia [8,9,10,11]. However, NE is a syndrome rather than a diagnosis. Indeed, several disorders can simulate or coexist with HIE, including metabolic disorders, genetic diseases, intracranial hemorrhage, stroke, central nervous system malformations, infections, and epileptic encephalopathies [12,13]. For this reason, the terms NE and HIE should not be used interchangeably [3]. Despite their clinical importance, HIE mimics frequently remain underdiagnosed in routine practice. Several factors contribute to this diagnostic gap. First, the clinical presentation of genetic and metabolic mimics can closely overlap with that of true hypoxic–ischaemic injury, including altered consciousness, hypotonia, respiratory depression, and neonatal seizures, making the two difficult to distinguish on clinical grounds alone [12,14,15]. Second, the diagnosis of HIE is often established acutely, based on perinatal history, Apgar scores, cord blood gases, and early clinical examination, in order to determine eligibility for therapeutic hypothermia within a narrow therapeutic window; this urgency leaves little room for a comprehensive etiological work-up before treatment decisions must be made [8,9,10,11]. Third, once a diagnosis of perinatal asphyxia or HIE has been assigned, subsequent clinical reasoning and investigations may remain anchored to this initial impression, potentially delaying recognition of an underlying genetic or metabolic disorder, particularly when neuroimaging findings are non-specific or overlap with hypoxic–ischaemic patterns [14,15]. Fourth, many neonatal genetic disorders present with an incomplete or evolving phenotype in the first days of life, and dysmorphic features, metabolic derangements, or seizure semiology suggestive of a specific genetic etiology may only become apparent after the acute neonatal period [16]. Finally, access to genomic sequencing remains uneven across neonatal units, and, where available, conventional sequencing approaches often have turnaround times that exceed the acute management window, further limiting their contribution to the initial diagnostic process [16,17]. As a result, HIE mimics are likely underrecognized in clinical practice, underscoring the need for a systematic evaluation of the role and timing of genomic sequencing in this population. Establishing the correct etiology is essential because prognosis, recurrence risk, treatment, and family counselling differ substantially among these conditions. The introduction of therapeutic hypothermia has improved outcomes in infants with moderate-to-severe HIE [8]. Nevertheless, the therapeutic window is limited to the first hours after birth, and treatment decisions often rely on clinical suspicion before the underlying diagnosis is fully established. Therefore, some newborns initially diagnosed with HIE are later found to have alternative genetic or metabolic disorders [16,18,19], whereas others may present with both hypoxic injury and an underlying genetic susceptibility. Distinguishing true HIE from genetic or metabolic mimics therefore remains an important clinical challenge. Advances in next-generation sequencing have rapidly expanded the diagnostic evaluation of neonatal neurological disorders. Whole-exome sequencing (WES), whole-genome sequencing (WGS), clinical exome sequencing (CES), and targeted gene panels are increasingly used in infants with unexplained NE or atypical HIE presentations [20,21]. Beyond conventional WES, WGS is increasingly favored in the neonatal setting because it captures non-coding and structural variants missed by exome-based approaches and can be performed as a rapid, trio-based test with turnaround times compatible with acute neonatal decision-making [22,23,24]. Long-read sequencing platforms are also emerging as a promising tool in critically ill neonates, offering ultra-rapid turnaround and improved detection of structural variants, repeat expansions, and complex rearrangements, although clinical experience in the NE population remains limited [25]. In addition, concurrent mitochondrial genomic sequencing performed alongside nuclear exome or genomic sequencing has enabled timely diagnosis of mitochondrial disorders presenting with encephalopathy and multiorgan involvement in the neonatal period [26]. Finally, RNA sequencing has been proposed as a complementary approach for neonates who remain undiagnosed after exome or genomic sequencing, improving diagnostic yield by clarifying the functional consequences of variants of uncertain significance and detecting aberrant splicing or expression not captured by DNA-based sequencing alone [27]. These technologies have identified pathogenic variants in genes involved in ion channel function, neurotransmission, neuromuscular disorders, metabolic pathways, mitochondrial diseases, and neurodevelopmental syndromes. In selected cohorts, genomic sequencing has provided clinically relevant diagnoses that would not have been recognized by conventional investigations alone [28,29]. Genetic results may adjust the initial diagnosis guiding targeted treatment and improving genetic counselling for affected families. Although several studies have investigated genomic sequencing in NE, the available evidence remains heterogeneous. Existing clinical recommendations increasingly support the use of genomic sequencing in critically ill neonates. The American College of Medical Genetics and Genomics (ACMG) has issued an evidence-based clinical guideline recommending that exome or genomic sequencing be offered as a first- or second-tier diagnostic test for pediatric patients, including neonates, with multiple congenital anomalies or a suspected genetic etiology, based on its superior diagnostic yield compared with traditional stepwise testing [30]. Similarly, an intersociety policy statement from Italian scientific societies has outlined criteria for the appropriate use of whole-exome sequencing in critically ill newborns, emphasizing multidisciplinary evaluation, informed consent, and structured reporting of clinically actionable findings [17]. In recent years, rapid trio genome and exome sequencing has become increasingly feasible within neonatal intensive care units, with turnaround times as short as 24–72 h in specialized centers [22,23]. This technological progress allows genomic results to become available within a clinically actionable window, potentially informing decisions regarding therapeutic hypothermia eligibility, treatment escalation, and family counselling during the acute neonatal period. Given this rapidly evolving landscape and the expanding clinical adoption of rapid genomic sequencing in critically ill neonates, a systematic evaluation of the diagnostic yield and clinical utility of genomic sequencing specifically in NE is particularly timely. Study populations differ considerably with respect to inclusion criteria, disease severity, timing of genomic sequencing, sequencing strategy, and outcome reporting. The aim of this systematic review was to assess the current evidence on genomic sequencing in NE. We reviewed studies using exome or genomic sequencing and other next-generation sequencing approaches to assess diagnostic yield, molecular findings, and the potential clinical impact of genomic sequencing in infants with NE, including HIE and its genetic mimics.

## 2. Materials and Methods

### 2.1. Protocol and Registration

This systematic review was conducted according to the Preferred Reporting Items for Systematic Reviews and Meta-Analyses (PRISMA 2020) statement and the Cochrane Handbook for Systematic Reviews of Interventions [31]. The review protocol was prospectively registered in the International Prospective Register of Systematic Reviews (PROSPERO; registration number: CRD420261428433).

### 2.2. Eligibility Criteria, Information Sources and Search Strategy

A systematic literature search was performed in PubMed/MEDLINE, Embase, and Scopus from database inception to June 2026, without language or publication date restrictions. The complete search strategies for each database are reported in Supplementary Table S1. The search combined terms related to neonatal encephalopathy (including “neonatal encephalopathy”, “hypoxic-ischaemic encephalopathy”, “hypoxic ischemic encephalopathy”, HIE, and “hypoxic-ischaemic brain injury”) with terms related to genomic sequencing (including “whole-exome sequencing”, “whole-genome sequencing”, WES, WGS, “next-generation sequencing”, NGS, exome, genome, sequencing, genetic, and genomic). Moreover, the review question was structured according to the PICO framework: (1) Population: neonates with NE, HIE, suspected HIE, HIE mimics, or unexplained NE; (2) Intervention: genomic sequencing, including WES, WGS, clinical exome sequencing (CES), rapid genomic sequencing, and other NGS-based diagnostic approaches; (3) Comparator: no comparator was required, although comparative data between genetically diagnosed and non-diagnosed patients were extracted when available; and (4) Outcomes: diagnostic yield of genomic sequencing and related clinical outcomes.

### 2.3. Selection of the Included Studies

Two reviewers independently screened titles and abstracts according to the predefined eligibility criteria. Potentially eligible studies underwent full-text review. Disagreements were resolved through discussion, and when consensus could not be reached, a third reviewer decided. Studies were considered eligible if they satisfied the following criteria: (i) included neonates (≤28 days of age) diagnosed with NE, suspected or confirmed HIE, HIE mimics, or unexplained NE; (ii) evaluated genomic sequencing for diagnostic purposes, including WES, WGS, CES, rapid genomic sequencing, or targeted NGS gene panels; (iii) reported at least one outcome related to diagnostic yield or molecular diagnosis; and (iv) were original human studies, including prospective or retrospective cohort studies, case–control studies, cross-sectional studies, or secondary analyses of prospective cohorts. Case reports, case series including fewer than five patients, conference abstracts, editorials, reviews, animal studies, and studies without original patient-level data were excluded. This threshold was chosen to reduce the risk of selection and publication bias inherent to very small case series and single case reports, which are more likely to describe atypical or highly selected presentations and do not allow for a meaningful assessment of diagnostic yield at the cohort level. This approach is consistent with common systematic review methodology for diagnostic yield studies When multiple publications reported overlapping cohorts, only the most comprehensive or recent report was included.

### 2.4. Data Extraction

Two independent reviewers performed the data extraction with a standardized data collection form. Extracted variables included study characteristics (first author, publication year, country, study design, study setting), patient demographics, eligibility criteria, clinical phenotype, definition of NE, HIE characteristics, therapeutic hypothermia, neuroimaging and neurophysiological findings, metabolic investigations, sequencing methodology, sequencing platform, gene panels, diagnostic yield, pathogenic and likely pathogenic variants, variants of uncertain significance, copy number variants, inheritance patterns, clinical management, genetic counselling, clinical outcomes, neurodevelopmental outcomes, mortality, and study limitations. Discrepancies were resolved by consensus. We also sought to extract data on variant classification criteria (e.g., adherence to ACMG/AMP guidelines), sequencing turnaround time, use of rapid versus standard sequencing protocols, singleton versus trio testing, and diagnostic laboratory accreditation status, given their potential influence on diagnostic yield [32]. However, these variables were inconsistently reported across the included studies and could therefore not be systematically extracted or compared.

### 2.5. Risk of Bias Assessment

Methodological quality was independently assessed by two reviewers using the Newcastle–Ottawa Scale (NOS) for observational studies [33]. The NOS assesses three domains: study selection, comparability, and outcome assessment, with an overall score ranging from 0 to 9. Studies scoring 7–9 points were considered at low risk of bias, those scoring 5–6 at moderate risk, and those scoring ≤4 at high risk of bias. Disagreements were resolved by discussion until consensus was reached.

### 2.6. Data Synthesis

A qualitative synthesis of the included studies was performed. Extracted data were summarized descriptively according to study characteristics, patient population, clinical phenotype, genomic sequencing strategy, diagnostic yield, molecular findings, and clinical implications. Given the substantial clinical and methodological heterogeneity across studies, including differences in patient selection, definitions of NE, sequencing approaches, variant interpretation, and reported outcomes, a quantitative meta-analysis was considered inappropriate. Therefore, pooled estimates of diagnostic yield were not calculated. Findings were synthesized narratively and presented in structured summary tables. Particular attention was given to the type of genomic sequencing performed, the spectrum of pathogenic and likely pathogenic variants identified, diagnostic yield, and the reported impact of genomic sequencing on diagnosis, clinical management, prognostic assessment, and genetic counselling. Studies investigating genetic susceptibility rather than diagnostic sequencing were included in the qualitative synthesis but were interpreted separately because of their different study objectives.

## 3. Results

### 3.1. Study Selection

**Figure 1:**
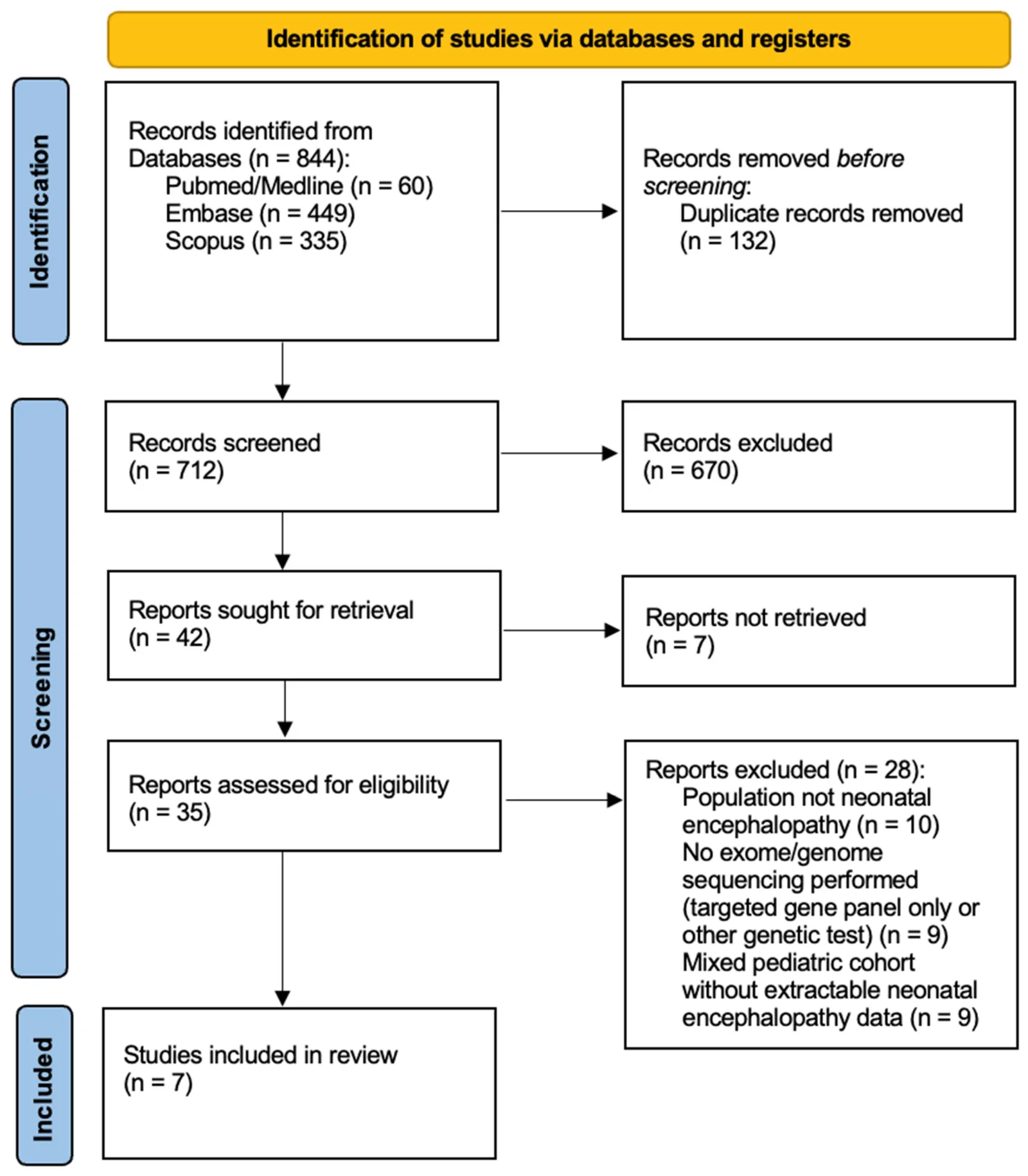
PRISMA 2020 flow diagram of the study selection process.

The systematic search identified 844 records. After removal of 132 duplicate records, 712 records underwent title and abstract screening, of which 670 were excluded. A total of 42 full-text articles were sought for retrieval; 7 reports could not be retrieved, leaving 35 studies for full-text eligibility assessment. Following full-text review, 28 articles were excluded. Ultimately, seven studies met the eligibility criteria and were included in the qualitative synthesis. This relatively small evidence base should be taken into account when interpreting the findings of this review, and the results should be regarded as preliminary rather than conclusive. No quantitative meta-analysis was performed because of substantial clinical and methodological heterogeneity among the included studies. The study selection process is summarized in Figure 1.

### 3.2. Risk of Bias Results

The methodological quality of the included studies was assessed using the Newcastle–Ottawa Scale (NOS). Table 1 shows the results. Overall, study quality was considered moderate: two studies reached a score of 7 [34,35], five studies of 5–6 [21,36,37,38,39], and no study was classified as low quality. The retrospective study design, the absence of comparison groups, the limited adjustment for confounding variables, and selection bias determined the overall moderate quality of the studies.

### 3.3. Characteristics of Included Studies

Table 2 shows the characteristics of the included studies. The studies were published between 2018 and 2025 and they were from Canada (n = 2) [35,36], the United States (n = 2) [21,34], China (n = 1), South Korea (n = 1) [38], and South Africa (n = 1) [37]. Study designs comprised two prospective cohort studies [35,36], two retrospective cohort studies [21,38], one observational cohort [39], one secondary analysis of a multicentre randomized controlled trial (HEAL) [34], and one multicentre case–control genetic association study [37]. The included studies involved highly heterogeneous neonatal populations, including unexplained NE, suspected or confirmed HIE, HIE mimics, and hypoxic brain injury.

### 3.4. Clinical Characteristics of the Study Populations

Table 3 summarizes the clinical characteristics of the included studies. The majority of the studies registered term or near-term neonates. Gestational ages ranged from around 36 to 39 weeks, instead birth weight varied according to the study population and inclusion criteria. Across studies, NE was characterized by combinations of altered consciousness, hypotonia, respiratory depression, feeding difficulties, abnormal reflexes, neonatal seizures, and abnormal neuroimaging findings. Therapeutic hypothermia was universally administered in cohorts specifically investigating moderate-to-severe HIE, whereas it was absent, not applicable, or inconsistently reported in studies primarily evaluating unexplained NE or HIE mimics. Neonatal seizures were among the most frequent presenting manifestations and were reported in the majority of included cohorts.

### 3.5. Genomic Sequencing Strategies and Molecular Findings

The genomic sequencing strategies and molecular findings are summarized in Table 4. Important heterogeneity was observed regarding genomic sequencing approaches, including WES, WGS, targeted NGS panels, and clinically indicated genomic sequencing performed according to individual patient characteristics. Between diagnostic sequencing studies, diagnostic yields ranged from 23.5% to 53.1%; however, these figures cannot be directly compared or pooled across studies. Several methodological factors account for this variability. Inclusion criteria differed considerably, ranging from neonates with unexplained encephalopathy without perinatal asphyxia to infants with suspected or confirmed HIE, which inherently affects the a priori likelihood of an underlying genetic diagnosis. Sequencing approaches also differed substantially, including trio-based versus singleton testing, WES versus WGS, and targeted gene panels versus genome-wide approaches, each with different sensitivity for single-nucleotide variants, structural variants, and non-coding variants. Some studies included candidate genes or variants of uncertain significance within their reported diagnostic yield, whereas others restricted their estimates to variants classified as pathogenic or likely pathogenic, which can artificially inflate or deflate the apparent yield depending on the threshold applied. The inclusion of copy number variants and chromosomal abnormalities in the diagnostic yield calculation was also inconsistent across studies. Finally, adherence to standardized variant interpretation frameworks, such as the ACMG/AMP guidelines, was not uniformly reported, and differences in variant classification standards between laboratories and studies may further contribute to the observed heterogeneity. The highest diagnostic yield was reported by Zhang et al. (53.1%) [39]; instead, Parobek et al., Bruun et al., Lee et al., and Ambrose et al. reported diagnostic yields ranging from approximately 24% to 36% [21,35,36,38]. On the other hand, Morell et al. reported clinically identified genetic abnormalities rather than systematic sequencing-based diagnostic yield [34]. Finally, Foden et al. explored genetic susceptibility using a case–control whole-genome sequencing approach and therefore did not report diagnostic yield [37].

Pathogenic and likely pathogenic variants involved a broad spectrum of genes associated with developmental and epileptic encephalopathies, neurodevelopmental disorders, metabolic diseases, mitochondrial disorders, and chromosomal abnormalities. Recurrently identified genes included SCN2A, KCNQ2, CACNA1A, STXBP1, PTPN11, BCOR, MMUT, COQ2, and GBE1. Copy number variants and chromosomal abnormalities were reported in several studies, while variants of uncertain significance are still common in studies employing broader sequencing strategies.

### 3.6. Clinical Utility and Patient Outcomes

Table 5 shows clinical implications of genomic sequencing. Through studies, genomic sequencing frequently resulted in revision or confirmation of the underlying diagnosis, particularly among neonates initially classified as having unexplained NE or suspected HIE. Several studies reported that molecular diagnoses influenced prognostic assessment, recurrence risk estimation, and genetic counselling. In selected cases, genetic findings also informed disease-specific management, including the use of sodium-channel blockers, lamotrigine, ketogenic diet, or metabolic therapies. These findings indicate that the clinical utility of genomic sequencing in this population extends beyond guiding disease-specific treatment. In several studies, establishing a molecular diagnosis effectively ended a prolonged diagnostic odyssey for the family, replacing a purely descriptive label of “unexplained neonatal encephalopathy” or “suspected HIE” with a defined genetic diagnosis, and informed reproductive counselling regarding recurrence risk in future pregnancies. However, none of the included studies systematically reported on other potential dimensions of clinical utility that have been described in the broader genomic medicine literature, such as the avoidance of unnecessary invasive or repeated investigations, the use of genetic results to inform palliative care and end-of-life decision-making, or eligibility for gene-specific clinical trials. These represent important outcome domains that should be systematically captured in future studies evaluating the clinical utility of genomic sequencing in NE. Reported clinical outcomes were heterogeneous across studies. Neurodevelopmental impairment was common among genetically diagnosed patients, with several cohorts reporting developmental delay, epilepsy, cerebral palsy, or severe intellectual disability during follow-up. Additionally, mortality was variable depending on the underlying genetic diagnosis and study population; in particular, it ranged from isolated neonatal deaths in small cohorts to higher mortality rates among genetically affected subgroups. Follow-up duration ranged from the neonatal hospitalization period to 2 years after birth.

## 4. Discussion

This systematic review summarizes the available evidence on genomic sequencing in neonatal encephalopathy. Overall, the included studies suggest that genomic sequencing can identify clinically relevant molecular diagnoses in a meaningful proportion of neonates with unexplained NE, suspected HIE, HIE mimics, or hypoxic brain injury. It should be emphasized from the outset that this evidence base remains limited, comprising only seven studies with heterogeneous designs and, in most cases, modest sample sizes. As such, the findings summarized below should be interpreted as hypothesis-generating rather than definitive, and larger, adequately powered studies are needed before firm clinical recommendations can be made. Diagnostic yield varied widely, from around 23% to more than 50% in diagnostic sequencing cohorts. This variability reveals differences in study design, patient selection, sequencing strategy, and variant interpretation. Despite this heterogeneity, the evidence consistently shows that genetic disorders represent an important and probably underrecognized component of NE. A key finding is that NE and HIE should not be treated as interchangeable entities. HIE remains the most common cause of NE and requires urgent recognition because therapeutic hypothermia must be started within a narrow therapeutic window. However, several genetic, metabolic, neuromuscular, epileptic, and syndromic disorders can mimic HIE or coexist with hypoxic–ischaemic injury [14,15,17]. This distinction is clinically relevant. A neonate may fulfil criteria for suspected HIE and still have an underlying genetic disorder [40,41]. On the other hand, a genetic disease may present low Apgar scores, respiratory depression, seizures, abnormal tone, and brain MRI abnormalities that resemble hypoxic injury [42].

The studies included in this review identified pathogenic or likely pathogenic variants in genes involved in several biological pathways. These included ion channels, synaptic function, mitochondrial metabolism, neuromuscular function, chromatin regulation, vascular biology, and broader neurodevelopmental processes. Recurrent or clinically important genes included SCN2A, KCNQ2, CACNA1A, SCN8A, STXBP1, GBE1, COQ2, MMUT, PTPN11, BCOR, and others. This spectrum supports the concept that NE is genetically heterogeneous [1,38,43]. The diagnostic value of sequencing was most evident in neonates with unexplained NE, neonatal seizures, atypical evolution, metabolic abnormalities, congenital anomalies, dysmorphic features, or poor concordance between the clinical history and the presumed hypoxic–ischaemic mechanism. In these scenarios, genomic assessment helped determine diagnoses that would have been difficult with standard investigations alone. This is important because many neonatal genetic disorders have incomplete phenotypes during the first days of life [44]. The clinical picture may evolve only after the acute neonatal period. Therefore, reliance on early clinical signs alone may delay or miss the correct diagnosis. Genomic results had potential clinical utility in several domains. First, they clarified the underlying diagnosis. This is especially relevant in neonates initially labelled as having suspected HIE or unexplained NE [39]. Second, molecular diagnosis supported prognostic assessment [45]. Several identified conditions were associated with severe epilepsy, developmental delay, cerebral palsy, intellectual disability, or early mortality [12,16,19]. Third, genetic findings supported family counselling and recurrence risk assessment. This is particularly important for autosomal recessive, X-linked, inherited dominant, and de novo disorders with parental mosaicism. In selected cases, genomic sequencing also influenced treatment [8,46]. Reported examples included sodium-channel blockers for channelopathies, lamotrigine for specific epilepsy-related genes, ketogenic diet for developmental and epileptic encephalopathies, and metabolic or supportive disease-specific interventions [47,48,49,50]. A molecular diagnosis can still reduce diagnostic uncertainty, avoid unnecessary investigations, and support clinical follow-up, even when no targeted therapy is available. An important and often underappreciated challenge in the clinical application of genomic sequencing in NE is the frequent identification of variants of uncertain significance (VUS). Several of the included studies reported VUS or candidate variants alongside confirmed pathogenic findings, reflecting the substantial proportion of neonatal genetic cases that remain diagnostically unresolved at initial testing. Interpretation of VUS in the acute neonatal period is particularly challenging because population-scale reference databases, functional evidence, and phenotype correlations are often more limited for rare, early-onset presentations than for later-onset disease, and computational prediction tools alone are frequently insufficient to reach a confident classification. VUS results also raise specific counselling challenges: parents must be informed that a variant of unknown significance does not currently establish a diagnosis, without either falsely reassuring them or generating undue anxiety, and clinicians must avoid altering acute management based on an unconfirmed finding. Related to this, genomic sequencing may also generate secondary or incidental findings unrelated to the indication for testing, and neonatal testing programs should define in advance whether and how such findings will be reported and consented for, in line with existing professional guidance [51]. Finally, because variant classification is not static, periodic reanalysis of previously uncertain or negative results is essential: reclassification driven by updated population databases, functional data, and evolving phenotypic correlation can convert VUS into pathogenic or likely pathogenic diagnoses over time. In one recent neonatal cohort, systematic reanalysis reclassified approximately 30% of VUS and improved overall diagnostic yield by nearly three percentage points [52], underscoring the importance of structured reanalysis pathways and mechanisms to recontact families as new evidence emerges [53]. The findings of this review should be interpreted within the broader evolution of neonatal genomic medicine. Beyond considerations of speed, the choice between whole-exome and whole-genome sequencing has direct implications for diagnostic yield. WGS avoids the hybridization and PCR-based capture steps required for WES library preparation, resulting in more uniform sequencing coverage and a substantially lower rate of low-quality or false-positive variant calls; in a direct comparison, WGS identified several hundred additional high-quality coding variants per sample that were missed by WES, with a markedly higher validation rate than WES-exclusive calls [54]. More importantly for NE, WGS captures structural variants, copy number variants, and non-coding and deep intronic variants that fall outside the regions targeted by exome capture, including pathogenic variants in genes such as RNU4ATAC that are frequently absent from standard exome capture kits. In a head-to-head comparison against targeted gene panels, WGS achieved a diagnostic yield of 41% compared with 24% for panel-based testing, capturing all panel-based diagnoses while identifying additional cases missed by conventional approaches [55]. WGS also enables a single comprehensive test that can be periodically reanalyzed as new gene-disease associations are discovered, without the need for repeat sequencing, an approach particularly relevant for neonates with unexplained encephalopathy in whom an initial negative result does not exclude a genetic etiology. These considerations support a preference for WGS over WES where resources allow, particularly for neonates with atypical, syndromic, or unexplained presentations in whom structural or non-coding pathogenic variants are more likely to be missed by exome-based approaches. Rapid exome and genomic sequencing are increasingly feasible in neonatal intensive care and are becoming part of standard practice in many high-resource NICUs. A recent systematic review reported a weighted average diagnostic yield of 37% (range 19–83%) with rapid genomic sequencing, rising to 48% for ultra-rapid genome sequencing (URGS) specifically, compared with 36% for standard rapid genome/exome sequencing and only 10% for chromosomal microarray [56]. Turnaround times have shortened considerably, with URGS providing provisional results in a median of approximately 36 h in routine clinical operation, and as fast as 20 h in optimized settings, while rapid genome and exome sequencing protocols typically return results within 10 days of sample receipt [56]. Rapid sequencing programs have also been associated with meaningful reductions in healthcare costs, further supporting their integration into critical care pathways [56]. These findings have direct implications for NE. The early management of suspected HIE cannot wait for complete etiological clarification; however, rapid genomic sequencing could be integrated into the diagnostic pathway after stabilization, or even during the first days of life in selected infants, allowing genomic results to inform management decisions within a clinically relevant timeframe. Additionally, rapid trio sequencing may be more informative than sequential single-gene tests or broad delayed investigations [57]. The consistent advantage of trio sequencing over singleton sequencing observed across the genomic medicine literature reflects several methodological mechanisms rather than a simple increase in the number of individuals sequenced. First, the availability of parental genotypes allows immediate determination of variant inheritance, enabling de novo variants to be identified and classified with high confidence according to the ACMG criterion PS2 (confirmed de novo), which is considerably stronger than the PM6 criterion used to infer a variant as “assumed de novo” in singleton analysis without parental confirmation [58]. Second, trio data allow phasing of compound heterozygous variants, distinguishing biallelic pathogenic combinations inherited in trans from variants inherited together in cis on the same parental allele, a distinction that cannot be reliably made from proband-only data. Third, inherited variants shared with unaffected parents can be filtered out, substantially reducing the number of variants requiring manual curation and the overall burden of variants of uncertain significance, while simultaneously increasing diagnostic yield [58,59]. Finally, trio analysis reduces reliance on additional confirmatory Sanger segregation testing and appears to partly compensate for variability in analyst experience, supporting more consistent variant interpretation across laboratories [58,59]. In studies directly comparing the two approaches, trio sequencing has been associated with modestly to substantially higher diagnostic yields than singleton sequencing, with the largest differences observed in prospective clinical settings [58,59]. These considerations reinforce the rationale for prioritizing trio-based sequencing whenever both parents are available, particularly in the acute neonatal setting where rapid and confident variant classification is essential. One included study investigated genetic susceptibility rather than individual molecular diagnosis [37]. This is conceptually different from diagnostic sequencing. It did not provide diagnostic yield, but it suggested that non-coding variants may influence susceptibility, severity, or response to hypoxic–ischaemic injury. This area is still exploratory. However, it is biologically plausible that genetic background may modify vulnerability to perinatal hypoxia, inflammation, mitochondrial dysfunction, vascular injury, or impaired recovery after cooling [17,21,38,41]. Future studies should distinguish clearly between genetic mimics of HIE and genetic modifiers of true hypoxic–ischaemic injury. This review has several strengths. It addresses a clinically relevant and emerging topic. It includes different genomic approaches and captures both diagnostic sequencing studies and studies focused on HIE-like presentations. It also summarizes clinical utility, not only diagnostic yield. The main limitation is the heterogeneity of the included studies. Populations differed substantially. Some cohorts included unexplained NE without perinatal asphyxia, whereas others focused on suspected HIE or hypoxic brain injury. Sequencing strategies also differed, including WES, WGS, targeted panels, and clinically indicated mixed testing. Variant classification was not uniform, and some studies included candidate genes or VUS in their broader diagnostic estimates. Follow-up was variable and often short. Moreover, several studies had small sample sizes and possible selection bias, because sequencing was often performed in highly selected neonates with severe, atypical, or unexplained phenotypes. Evidence on the economic impact of genomic sequencing specifically in NE also remains limited. None of the included studies systematically reported healthcare costs, NICU length of stay, or reductions in downstream confirmatory or exploratory testing (e.g., metabolic work-up, neuroimaging, or sequential single-gene testing) attributable to genomic sequencing. Cost-effectiveness data from broader critically ill infant populations, including infants with congenital anomalies and multisystem disease, suggest that rapid genomic sequencing can reduce length of stay and net healthcare costs when it changes management [60]; however, these findings cannot be directly extrapolated to neonates with encephalopathy or suspected HIE, a population with distinct diagnostic pathways, therapeutic hypothermia protocols, and resource utilization patterns. Dedicated economic evaluations, including formal cost-effectiveness and cost-utility analyses, are therefore needed to determine the value of genomic sequencing specifically within the NE care pathway. For these reasons, pooled diagnostic yield was not calculated. Furthermore, although our search strategy combined broad terms related to NE, HIE, and genomic sequencing, it may not have fully captured studies describing neonatal seizures, neonatal epileptic encephalopathy, or metabolic encephalopathy that did not explicitly use the term “neonatal encephalopathy” or its synonyms, potentially leading to an underestimation of the full body of relevant literature. In addition, our decision to exclude case series with fewer than five patients, while intended to minimize selection and publication bias, may have excluded early reports describing novel or rare genetic causes of NE identified in small cohorts, some of which have historically contributed important insights into emerging genetic disorders in this field. Variability in diagnostic yield across studies may also be partly explained by differences in variant classification criteria, sequencing turnaround time, use of rapid versus standard protocols, singleton versus trio testing strategies, and diagnostic laboratory accreditation, all of which are known to influence diagnostic outcomes in genomic sequencing. However, these variables were not consistently reported across the included studies, precluding a systematic comparison. Future studies should use standardized definitions of NE, HIE, suspected HIE, and HIE mimics. They should report gestational age, perinatal criteria, therapeutic hypothermia, MRI patterns, EEG findings, metabolic testing, sequencing timing, family structure, and variant classification in a uniform way. Larger prospective multicentre studies are needed. Ideally, these should evaluate rapid trio WES or WGS in predefined subgroups of neonates with NE. They should also assess clinical utility, time to diagnosis, cost-effectiveness, parental counselling, and long-term neurodevelopmental outcomes. In clinical practice, genomic sequencing should not replace careful neurological assessment, EEG, MRI, metabolic testing, and evaluation for infection or stroke. Instead, it should complement them.

## 5. Conclusions

Genomic sequencing has an important role in the etiological evaluation of NE. It can identify HIE mimics, reveal coexisting genetic disorders, support precision medicine, and improve counselling. Current evidence remains limited, comprising only seven studies with substantial heterogeneity, but it supports a shift toward earlier and more structured use of genomic sequencing in selected neonates with NE, pending confirmation in larger prospective studies.

## Figures and Tables

**Table 1:** Risk of bias assessment of the included studies using the Newcastle–Ottawa Scale (NOS).

Author	Year	Selection (Max 4)	Comparability (Max 2)	Outcome (Max 3)	Total NOS (/9)	Risk of Bias
Foden [37]	2025	★★★☆	★☆	★☆☆	5/9	Moderate–high
Ambrose [36]	2025	★★★★	☆☆	★☆☆	5/9	Moderate
Zhang [39]	2024	★★★☆	☆☆	★★☆	5/9	Moderate
Parobek [21]	2024	★★★☆	☆☆	★★★	6/9	Moderate
Morell [34]	2024	★★★☆	★☆	★★★	7/9	Moderate
Lee [38]	2024	★★★	★☆	★★☆	6/9	Moderate
Bruun [35]	2018	★★★★	☆☆	★☆★	7/9	Moderate

Notes: NOS, Newcastle-Ottawa Scale. Maximum of 4 stars for Selection, 2 for Comparability, and 3 for Outcome (total range 0–9).★ = point awarded; ☆ = point not awarded. Risk of bias: low = 7–9 points; moderate = 5–6 points; high = ≤4 points.

**Table 2:** Characteristics of the included studies.

Author	Year	Country	Study Design	Population	Sample Size
Foden [37]	2025	South Africa	Multicentre case–control genetic association study	Neonates ≥ 36 weeks with moderate-to-severe NESHIE undergoing therapeutic hypothermia	172 cases; 288 controls
Ambrose [36]	2025	Canada	Prospective cohort	Term neonates with NE with or without seizures	17
Zhang [39]	2024	China	Observational cohort	Neonates with unexplained NE	113
Parobek [21]	2024	USA	Retrospective cohort	Neonates ≥ 35 weeks with suspected or perinatal HIE undergoing exome/genomic sequencing	24
Morell [34]	2024	USA	Secondary analysis of a multicentre randomized controlled trial (HEAL)	Infants with moderate-to-severe HIE undergoing therapeutic hypothermia	500
Lee [38]	2024	South Korea	Retrospective cohort	Neonates with NE accompanied by hypoxic brain damage who underwent targeted gene panel sequencing	34
Bruun [35]	2018	Canada	Prospective cohort	Term neonates with NE and/or seizures or abnormal brain MRI without perinatal asphyxia	19 enrolled (14 WES)

Abbreviations: HIE, hypoxic–ischaemic encephalopathy; NE, neonatal encephalopathy; NESHIE, neonatal encephalopathy suspected due to hypoxic–ischaemic encephalopathy.

**Table 3:** Clinical characteristics of the included study populations.

Author	Year	Term Infants	Gestational Age	Birth Weight	Clinical Phenotype	Therapeutic Hypothermia	Neonatal Seizures
Foden [37]	2025	Yes	≥36 weeks	≥1800 g	Moderate-to-severe NESHIE undergoing TH	All patients	Inclusion criterion ‡
Ambrose [36]	2025	Yes	≥37 weeks	NR	Term NE with abnormal consciousness, respiratory depression, hypotonia, and/or seizures	NR	14/17
Zhang [39]	2024	Predominantly term infants	38.6 ± 1.5 weeks	2957 ± 561 g	Unexplained NE with seizures, hypotonia, respiratory distress, and metabolic abnormalities	NR	Frequent
Parobek [21]	2024	Yes	≥35 weeks	NR	Suspected/perinatal HIE with NE	15/24	Reported (exact frequency NR)
Morell [34]	2024	Yes	≥36 weeks	≥1800 g (inclusion criterion)	Moderate-to-severe HIE	All patients	EEG seizures: 33% vs. 22% *
Lee [38]	2024	No †	NR	NR overall; 2836 ± 588 g vs. 3126 ± 551 g *	NE with hypoxic brain injury, seizures, and respiratory impairment	4/34	28/34
Bruun [35]	2018	Yes	≥37 weeks	NR	NE without perinatal asphyxia, presenting with seizures, hypotonia, poor feeding, apnea, and abnormal EEG/MRI	NA	14/14

Abbreviations: EEG, electroencephalography; HIE, hypoxic–ischaemic encephalopathy; MRI, magnetic resonance imaging; NA, not applicable; NE, neonatal encephalopathy; NESHIE, neonatal encephalopathy suspected due to hypoxic–ischaemic encephalopathy; NR, not reported. * Values refer to comparisons between infants with and without genetic or congenital anomalies and do not represent the overall study population. † Moderate/late preterm infants were included; only infants < 32 weeks’ gestation were excluded. ‡ Seizures were part of the inclusion criteria but were not mandatory; the exact prevalence was not reported.

**Table 4:** Genomic sequencing strategies and molecular findings of the included studies.

Author	Year	Genetic Test	Family Design	Patients Sequenced (n)	Diagnostic Yield	P/LP Variants	VUS	CNVs	Main Genes Identified
Foden [37]	2025	WGS (genetic association study)	Unrelated cases	172 cases (WGS); 288 controls	NA	NA	NA	NA	*PRKN*, *SLCO3A1*, *ZGRF1*, *CNTN5*, *ASXL2*, *PADI4*, *ADAMTS3*, among others
Ambrose [36]	2025	Trio GS	Trio	17	4/17 (23.5%) confirmed diagnoses; 7/17 (41.2%) including candidate genes	4 P/LP diagnoses (PPP2R5D, BCOR, CFL2, SCN2A)	Candidate variants in DST, STAB2, SORCS2, CTNND2, CELF4, and ASTN1	CNVs involving CELF4 and ASTN1	*PPP2R5D*, *BCOR*, *CFL2*, *SCN2A*, *DST*, *SORCS2*, *STAB2*, *CTNND2*, *CELF4*, *ASTN1*
Zhang [39]	2024	WES or targeted NGS panel	Family-based	113	60/113 (53.1%)	9 P/LP variants in novel-variant subgroup	6 VUS in novel-variant subgroup	8 CNVs + 4 chromosomal abnormalities	*CACNA1G*, *CHD4*, *HNRNPK*, *COQ2*, *MMUT*, *CPS1*, *KCNQ2*, among others
Parobek [21]	2024	Clinical ES/WGS	Proband/trio	23 ES; 1 GS	6/24 (25.0%)	6 P/LP diagnoses	6 indeterminate findings	One 15q13.3 deletion	*KIF1A*, *GBE1*, *ACTA1*, *DUOX2*, *PTPN11* deletion
Morell [34]	2024	Clinically indicated testing (WES, SNP array, karyotype or targeted testing)	NR	NR	15/500 (3.0%) genetic abnormalities †	NR	NR	Chromosomal abnormalities reported	*PTPN11*, *COL2A1*, *KIAA1109*, *JAG1*, *SLC6A5*, *G6PD*, *CYBB*, *TSC1*, *NF1*, *SHOX*, among others
Lee [38]	2024	Targeted NGS panel	Singletons; trio confirmation in 4 cases	34	11/34 (32.4%)	11 P/LP diagnoses (9 genes)	NR	None reported	*CACNA1A*, *KCNQ2*, *SCN2A*, *SCN8A*, *STXBP1*, *NSD1*, *PURA*, *ZBTB20*, *ENG*
Bruun [35]	2018	Trio WES	Trio	14	5/14 (35.7%)	5 confirmed molecular diagnoses	Candidate variants (RAI1, AACS)	None	*SCN2A*, *KCNQ2*, *GNAO1*, *LIAS*, *CUL4B*

Abbreviations: CNV, copy number variant; ES, exome sequencing; GS, genomic sequencing; NA, not applicable; NGS, next-generation sequencing; NR, not reported; P/LP, pathogenic/likely pathogenic; VUS, variant of uncertain significance; WES, whole-exome sequencing; WGS, whole-genome sequencing. Diagnostic yield was calculated according to each study definition and is therefore not directly comparable across studies because of differences in sequencing strategy, variant interpretation, and inclusion of candidate genes or copy number variants. Diagnostic yield should be interpreted cautiously because sequencing strategies, inclusion criteria, and variant classification differed substantially across studies. † Morell et al. reported clinically identified genetic abnormalities rather than a sequencing-based diagnostic yield, as genomic sequencing was performed only when clinically indicated.

**Table 5:** Clinical utility and outcomes of genomic sequencing in neonatal encephalopathy.

Author	Year	Change in Diagnosis	Impact on Clinical Management	Genetic Counselling	Outcome	Neurodevelopmental Outcome	Mortality	Follow-Up
Foden [37]	2025	No diagnostic reclassification	No immediate clinical impact; potential future risk stratification	Not reported	60% clinically improved during hospitalization	NR	4/172	Hospital stay
Ambrose [36]	2025	Established molecular diagnoses and expanded disease phenotypes	Potential implications for prognosis and management	Yes	Variable clinical outcomes	GDD, CP, epilepsy, and cognitive dysfunction in several survivors	3/17	Variable (neonatal period to several years)
Zhang [39]	2024	Molecular diagnosis established in unexplained NE	Potential implications for treatment optimization and metabolic/antiepileptic management	NR/possible	Variable	Neurodevelopmental delay in several survivors	6/15 in novel variant subgroup	Variable; up to 12 months
Parobek [21]	2024	Reclassified several presumed HIE cases as genetic disorders	Influenced diagnosis, prognosis and recurrence risk	Yes	Variable	NR	6/24 (1 with genetic diagnosis; 5 without)	First year of life
Morell [34]	2024	Previously unsuspected genetic/congenital anomalies identified	Mainly prognostic implications	NR	Death or NDI: 75% vs. 50%	Worse BSID-III scores, higher CP rate, and GMFCS > 1	3/24	2 years
Lee [38]	2024	Identified genetic etiologies despite hypoxic brain injury	Precision therapies (sodium-channel blockers, lamotrigine, ketogenic diet)	Yes	Persistent epilepsy and severe neurological disability common	Moderate-to-profound intellectual disability common	1/34	≥1 year
Bruun [35]	2018	Molecular diagnosis established in unexplained NE/HIE mimics	Supported targeted diagnosis and informed future targeted therapies	Yes	Variable	Developmental delay in several cases	1/14	NR

Abbreviations: BSID-III, Bayley Scales of Infant and Toddler Development, Third Edition; CP, cerebral palsy; GDD, global developmental delay; HIE, hypoxic–ischaemic encephalopathy; NDI, neurodevelopmental impairment; NE, neonatal encephalopathy; NR, not reported.

## Data Availability

No new data were created or analyzed in this study. Data sharing is not applicable to this article.

## Supplementary Materials

The following supporting information can be downloaded at: https://www.mdpi.com/article/10.3390/genes17080862/s1, Table S1: Literature search strategy; Table S2: PRISMA 2020 checklist [31].

## Abbreviations

The following abbreviations are used in this manuscript:

BSID-III	Bayley Scales of Infant and Toddler Development, Third Edition
CES	Clinical Exome Sequencing
CNV	Copy Number Variant
CP	Cerebral Palsy
EEG	Electroencephalography
ES	Exome Sequencing
GDD	Global Developmental Delay
GS	Genomic sequencing
HEAL	High-Dose Erythropoietin for Asphyxia and Encephalopathy (clinical trial)
HIE	Hypoxic–Ischaemic Encephalopathy
MRI	Magnetic Resonance Imaging
NA	Not Applicable
NE	Neonatal Encephalopathy
NESHIE	Neonatal Encephalopathy Suspected due to Hypoxic–Ischaemic Encephalopathy
NDI	Neurodevelopmental Impairment
NGS	Next-Generation Sequencing
NICU	Neonatal Intensive Care Unit
NOS	Newcastle–Ottawa Scale
NR	Not Reported
P/LP	Pathogenic/Likely Pathogenic
PICO	Population, Intervention, Comparator, Outcome
PRISMA	Preferred Reporting Items for Systematic Reviews and Meta-Analyses
PROSPERO	International Prospective Register of Systematic Reviews
SNP	Single Nucleotide Polymorphism
VUS	Variant of Uncertain Significance
WES	Whole-Exome Sequencing
WGS	Whole-Genome Sequencing
