# Supplementary material for: Genomic Sequencing in Neonatal Encephalopathy and Suspected Hypoxic–Ischaemic Encephalopathy: A Systematic Review
Source: Genes (Basel). 2026 Jul 24;17(8):862. doi: 10.3390/genes17080862 (PMC13511764; doi:10.3390/genes17080862)
Supplement: Supplementary file 1 [file genes-17-00862-s001.zip › Supplementary Table S1 .pdf]

Supplementary Table S1. Literature search strategy

Database	Search strategy
**PubMed/MEDLINE**	("neonatal encephalopathy"[Title/Abstract] OR "hypoxic ischemic encephalopathy"[Title/Abstract] OR HIE[Title/Abstract] OR "hypoxic-ischaemic encephalopathy"[Title/Abstract] OR "hypoxic ischemic brain injury"[Title/Abstract] OR "hypoxic-ischemic brain injury"[Title/Abstract]) AND ("whole exome sequencing"[Title/Abstract] OR "whole genome sequencing"[Title/Abstract] OR exome[Title/Abstract] OR genome[Title/Abstract] OR WES[Title/Abstract] OR WGS[Title/Abstract] OR "next generation sequencing"[Title/Abstract] OR NGS[Title/Abstract] OR sequencing[Title/Abstract] OR genetic*[Title/Abstract] OR genomic*[Title/Abstract])
**Embase**	('neonatal encephalopathy'/exp OR 'hypoxic ischemic encephalopathy'/exp OR 'hypoxic ischemic brain injury'/exp OR 'neonatal encephalopathy':ti,ab OR 'hypoxic ischemic encephalopathy':ti,ab OR 'hypoxic ischaemic encephalopathy':ti,ab OR hie:ti,ab) AND ('whole exome sequencing'/exp OR 'whole genome sequencing'/exp OR 'next generation sequencing'/exp OR exome:ti,ab OR genome:ti,ab OR wes:ti,ab OR wgs:ti,ab OR sequencing:ti,ab OR genetic*:ti,ab OR genomic*:ti,ab)
**Scopus**	TITLE-ABS(("neonatal encephalopathy" OR "hypoxic ischemic encephalopathy" OR "hypoxic-ischaemic encephalopathy" OR HIE OR "hypoxic ischemic brain injury")) AND TITLE-ABS(("whole exome sequencing" OR "whole genome sequencing" OR WES OR WGS OR exome OR genome OR "next generation sequencing" OR NGS OR sequencing OR genetic* OR genomic*))

No language or publication date restrictions were applied during the electronic database searches. Duplicate records were removed before screening. Reference lists of all included studies and relevant reviews were manually screened to identify additional eligible articles.
